# Large Scale Comparative Codon-Pair Context Analysis Unveils General Rules that Fine-Tune Evolution of mRNA Primary Structure

**DOI:** 10.1371/journal.pone.0000847

**Published:** 2007-09-05

**Authors:** Gabriela Moura, Miguel Pinheiro, Joel Arrais, Ana Cristina Gomes, Laura Carreto, Adelaide Freitas, José L. Oliveira, Manuel A. S. Santos

**Affiliations:** 1 Department of Biology, Center for Environmental and Marine Studies, University of Aveiro, Aveiro, Portugal; 2 Institute of Electronics and Telematics Engineering, University of Aveiro, Aveiro, Portugal; 3 Department of Mathematics, University of Aveiro, Aveiro, Portugal; Temasek Life Sciences Laboratory, Singapore

## Abstract

**Background:**

Codon usage and codon-pair context are important gene primary structure features that influence mRNA decoding fidelity. In order to identify general rules that shape codon-pair context and minimize mRNA decoding error, we have carried out a large scale comparative codon-pair context analysis of 119 fully sequenced genomes.

**Methodologies/Principal Findings:**

We have developed mathematical and software tools for large scale comparative codon-pair context analysis. These methodologies unveiled general and species specific codon-pair context rules that govern evolution of mRNAs in the 3 domains of life. We show that evolution of bacterial and archeal mRNA primary structure is mainly dependent on constraints imposed by the translational machinery, while in eukaryotes DNA methylation and tri-nucleotide repeats impose strong biases on codon-pair context.

**Conclusions:**

The data highlight fundamental differences between prokaryotic and eukaryotic mRNA decoding rules, which are partially independent of codon usage.

## Introduction

A myriad of evolutionary forces shape the primary structure of coding components (ORFs) of genomes, herein called ORFeomes. These include genome and gene duplication, chromosome rearrangements, DNA recombination, deletions and insertions, transposition of mobile elements, single nucleotide polymorphisms, nucleotide repeats and biased G+C pressure [1]–[4]. Apart from these DNA replication derived phenomena others arising from DNA transcription, mRNA stability and translation [5]–[7] are also likely to fine tune ORFeomes' primary structure, but their significance is not yet fully understood.

At the mRNA translation level, synonymous codon usage and codon-pair context (representing the pair of codons located in the A and P- ribosome sites) are expected to be under selective pressure since they affect mRNA decoding speed and accuracy [8]–[15]. Synonymous codon usage biases are explained mainly by G+C content and only secondarily by constraints imposed by mRNA translation variables [4], namely tRNA abundance, efficiency of tRNA charging, mRNA decoding efficiency (speed plus accuracy), mRNA stability and structure, gene expression, and amino acid composition [7], [13], [16]–[18]. The nucleotides surrounding a codon also influence synonymous codon usage, with the strongest influence arising from the interplay between the last nucleotide of a codon and the first nucleotide of the neighbor codon (N_1_N_2_ **N_3_** **N_1_**N_2_N_3_), the so called N_3_-N_1_ context [7], [19], [20]. Conversely to codon usage, the forces that modulate codon-pair context, with the exception of the context of initiation and termination codons [16], [21], are still poorly understood. The few studies carried out to date show, however, that codon-pair context has a direct impact on missense, nonsense and frameshifting errors [15], [22], [23].

In *E. coli*, missense error *in vivo*, under standard growth conditions, is in the order of 10^−3^ to 10^−4^ per codon decoded [24], [25]. Frameshifting and stop codon readthrough errors happen at levels of 3×10^−4^ to 10^−5^ and of 10^−3^ to 10^−6^, respectively [26], [27]. Under stress, namely amino acid starvation, these basal error rates increase significantly [16], indicating that decoding error in nature may be significantly higher than in optimal laboratory conditions. Furthermore, 30% of the newly synthesized proteins in HeLa, lymph node, L-K^b^ and dendritic cells are defective ribosomal products (DRiPs) that arise from missense, frameshifting and ribosome drop off at mRNA pausing sites [28]. Since protein synthesis utilizes 45% of the cell ATP, 30% DRiP rate represents 11% of wastage of total cellular energy [28]. Whether this is a common trend in all type of cells is unknown, however, peptides resulting from proteasome degradation of DRiPs are a major source of peptides for MHC class I molecules, highlighting an unanticipated role of mistranslation in immune cells [28].

It is not yet clear whether the ribosome drops off randomly or preferentially at specific mRNA drop off hot spots. In other words, it is important to elucidate whether average decoding error (10^−4^ to 10^−5^) is evenly distributed along mRNAs (average error) or whether it fluctuates along the mRNA? If so, how can decoding error hot spots be identified? In order to obtain insight into these questions and identify mRNA primary structural features that influence mRNA decoding error, we have developed a software package, statistical and graphical tools to study codon-pairs corresponding to ribosomal A- and P-site codons, using genome wide approaches (ANACONDA vs 1.0) [20], [29]. ANACONDA 1.0 already allowed us to demonstrate that codon-pair context is weakly modulated by G+C pressure [20]. In the present study, we have significantly improved ANACONDA (creating its version 2.0) and used it to carry out large scale comparative codon-pair context analysis using complete ORFeome sequences of 81 Eubacteria, 18 Archaea and 20 Eukaryota. The data show that i) codon-pair context is species specific, ii) there are general rules governing its evolution in the three domains of life and iii) in eubacteria and archeae codon-pair context is mainly determined by constraints imposed by the translational machinery, while, iv) in eukaryotes the emergence of DNA methylation and tri-nucleotide repeats influenced codon-pair context. The data suggests the existence of fundamental differences between prokaryotic and eukaryotic mRNA decoding rules and shows that codon-pair context is partially independent of codon usage.

## Results

### New tools for large scale comparative codon-pair context analysis

**Figure 1:**
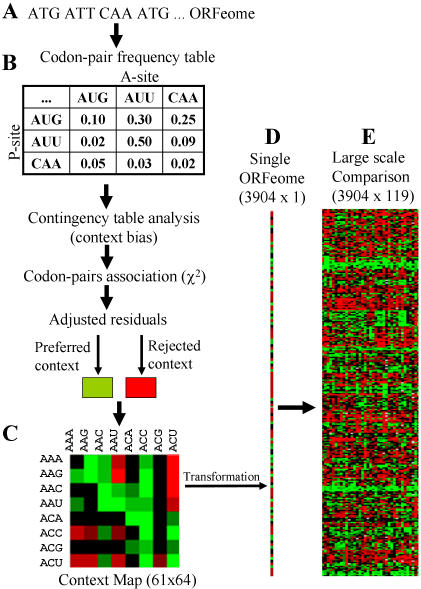
Flowchart of the codon-pair context analysis performed by ANACONDA. A) The software selects valid ORFs from the total set available for each species (ORFeome) and counts all combinations of two consecutive codons (codon-pair context) that are present in the sequences. B) The observed values are incorporated into a contingency table in which the lines correspond to the 5′ codon (ribosome P-site) and the columns to the 3′ codon (ribosome A-site) of each pair. C) The contingency table of observed values is then compared to another table in which the values expected under independence are calculated. The cell corresponding to each pair of codons was colored in green for preferred contexts or red for rejected ones. This produces a color-coded map for the 61×64 two-codon contexts of one ORFeome. D) To aid simultaneous comparison of a large set of ORFeomes the 61×64 map is automatically converted into one single column with 3904 lines, one for each pair of codons. E) Finally, the columns that illustrate the two-codon context bias of each individual ORFeome are placed side by side, yielding a large-scale codon context comparison map. Both maps for codon context bias, i.e. the 61×64 map for a single species and the large-scale codon context comparison map can be rearranged using clustering methodologies that highlight similar codon-pair context patterns. For detailed description of statistics and software, see Methods or [20], [29].

**Figure 2 pone-0000847-g002:**
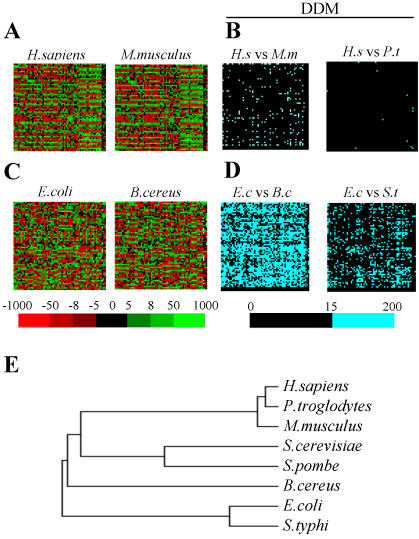
Codon-pair context is species specific. A) Individual codon-pair context maps built for various genomes followed phylogeny indicating that codon-pair context is species specific. For instance, the human ORFeome map is more similar to that of chimpanzee (*Pan troglodytes*) than to the mouse (*Mus musculus*) map. B) This result was confirmed using differential display maps (DDM) that subtract two codon-pair context maps. For example, *H. sapiens*–*M. musculus* (*H.s* vs *M.m*); *H. sapiens*–*P. troglodytes* (*H.s* vs *P.t*). In these differential display maps major codon-pair context differences (above 15) are shown in light blue and darker maps correspond to species with more similar codon-pair context biases. In the present example, the maps of *H.s* vs *M.m* and *H.s* vs *P.t* have 6% and 1% of blue cells, respectively. C and D) The same phylogenetical relationship could be detected for bacterial ORFeomes, as exemplified for *Escherichia coli*, *Bacillus cereus* and *Salmonella typhi*. The DDM built with these species have 55% (*E.c* vs *B.c*) and 20% (*E.c* vs *S.t*) of blue cells. E) Finally, the phylogenetical relationship was maintained when the above species were clustered according to the similarities of the codon-pair context maps. The yeasts *Saccharomyces cerevisiae* and *Schizosaccharomyces pombe* were added to include an intermediate group of lower eukaryotes in the tree. Adjusted residuals are colored in the maps according to the color scale shown, so that green cells correspond to preferred and red cells to rejected contexts.

The ANACONDA 1.0 algorithm developed previously [20], [29] simulates the ribosome during decoding by reading Open Reading Frames (ORFs) sequences, starting at the AUG initiation codon and moving the reading window three nucleotides at a time (Figure 1A). While doing this, it memorizes all codon-pairs, which represent A- and P-site codons during mRNA decoding. It then builds a codon-pair contingency table (Figure 1B) that renders itself to statistical analysis and permits determination of the codon-pair context bias [20]. The existence of association between codon-pairs is determined through the chi-square (χ^2^) test of independence and preferred and rejected pairs of codons are identified through the analysis of adjusted residuals for contingency tables. These rejected and preferred pairs of codons are then displayed in a 61x64 green (preferred) and red (rejected) color coded map that generates a global view of the codon-pair context data for any ORFeome (Figure 1C). ANACONDA 1.0 also clusters the data according to the context preferences and rejections (residuals values) and builds Differential Display Maps (DDM), which represent codon-pair context differences between two different ORFeomes (Figure 2).

In an attempt to identify putative general rules that govern codon-pair context, we have carried out large scale codon-pair comparisons, using ANACONDA version 2.0. For this, new algorithms and tools were developed to convert the 61x64 codon-pair context colour-coded maps into a single colour-coded column containing 3904 lines, representing all possible combinations of pairs of the 64 codons (Figure 1D). ANACONDA 2.0 compared these colour-coded columns, clustered the data and highlighted groups of codons that had similar pair preference and rejection patterns (Figures 1E–[Fig pone-0000847-g002] 3). Since the size of ORFeomes varied significantly between bacteria and eukaryotes, ANACONDA 2.0 normalized the data using the biggest ORFeome as a reference data set (Figure S1). This permitted carrying out direct comparisons of large and small ORFeomes and allowed the study of codon-pair context preferences (positive residual value; green color in the map) and rejections (negative residual values; red color in the map) of 119 ORFeomes of Eubacteria, Archaea and Eukarya, including the human and chimpanzee ORFeomes (Figure S2A–[Supplementary-material pone.0000847.s002] C).

### Codon-pair context preferences are species specific

Codon-pair context maps showed remarkable diversity from bacteria to high eukaryotes (Figure 2; Figure S3A–[Supplementary-material pone.0000847.s007] [Supplementary-material pone.0000847.s008] [Supplementary-material pone.0000847.s009] [Supplementary-material pone.0000847.s010] [Supplementary-material pone.0000847.s011] [Supplementary-material pone.0000847.s012] [Supplementary-material pone.0000847.s013] J). For example, codon-pair context preferences of the human (*Homo sapiens* or *H.s*) and mouse (*Mus musculus* or *M.m*) ORFeomes showed several differences, which were unveiled by direct comparison of ORFeomes and construction of Differential Display Maps (DDM) (Figure 2A,B), as described in our previous study [20]. Conversely, the codon-pair context maps for the chimpanzee (*Pan troglodytes* or *P.t*) and human ORFeomes were remarkably similar (Figure 2B), which was in agreement with the high homogeneity found for codon-pair distributions of both ORFeomes (data not shown). The same trend was found in bacteria. Indeed, the *Escherichia coli* (*E.c*) ORFeome codon-pair context map was more similar to that of *Salmonella typhi* (*S.t*) than to *Bacillus cereus* (*B.c* in Figure 2C,D). Clustering of the codon-pair context maps showed that codon-pair context follows rRNA phylogeny (Figure 2E), highlighting the possibility of using codon-pair context maps as species specific fingerprints. Furthermore, the overall correlation between the 3 domains of life was lower than that calculated within each domain, as the Spearman's correlations of the ranks (Table S1) showed low correlation coefficients between species of different domains of life, i.e. 0,452 for Eukarya vs Archaea; 0,450 for Eukarya vs Eubacteria; and 0,500 for Archaea vs Eubacteria. While correlation coefficients calculated between species of the same domain were high, i.e. 0,988 among Eukarya (between *H. sapiens* and *P. troglodytes*); 0,823 among Archaea (between *P. abyssi* and *P. horikoshii*); and 0,959 among Eubacteria (between *E. coli* and *S. flexneri*).

The distribution of residual values over the entire set of ORFeomes showed that the 3 domains of life have significantly different codon-pair preferences (Tables 1, 2). For example, codon-pair contexts with highest and lowest adjusted residual values showed no common codon-pairs in the 3 domains of life, suggesting fundamental differences between eukarya, eubacteria and archeae in codon-pair rules and in the evolutionary forces that shape ORFeomes primary structure. Interestingly, 9 out of the 10 codon-pair contexts with highest residual values (best codon-pairs) of all eukaryotic ORFeomes were pairs formed by identical codons (codon repeats) (Table 1). The same trend was also detected when the most frequently preferred codon-pair contexts for each domain were compared (Table 2). With this approach, common codon-pair contexts were identified for the 3 domains of life. For example, AAU-CCA and GGC-UGU had positive residuals in Eubacteria and Archaea. In Eukarya and Archaea ACU-AAG had negative residuals and AGA-AGA had positive residuals in Eubacteria and Eukarya. This suggested that, despite the species specificity of codon-pair context maps, at least some of the evolutionary constraints that shaped codon-pair context are conserved across species in the three domains of life.

**Table 1:** The most biased codon-pair contexts.

The 10 lowest residual values					
EUBACTERIA		ARCHAEA		EUKARYOTA	
Context	Residual	Context	Residual	Context	Residual
GCC>CUG	−308,976	GAC>GUC	−216,464	CUG>GAG	−135,197
CUG>GCG	−277,801	GGC>GCC	−201,205	GUC>GAG	−125,758
CUG>GGC	−248,528	CUG>GAG	−187,918	UUU>AAG	−118,366
UUC>GAG	−235,436	GGC>GCC	−183,471	AAU>UUA	−118,201
GCC>GGC	−231,399	CUC>GAG	−178,679	GGC>CUG	−110,765
CUG>CUC	−226,625	**CUC>GAG**	−176,574	CUC>GAG	−109,220
GUG>GCG	−224,022	GAG>CUC	−169,707	GCC>GAA	−107,698
CUC>CGC	−223,365	CUC>GAG	−148,041	CUC>CUG	−107,332
CUG>CAG	−222,711	UUA>GAU	−145,409	GCC>GAA	−107,194
GCC>CUG	−222,703	GAU>CCA	−141,241	AAA>UUU	−106,245
**The 10 highest residual values**					
CGA>UCG	921,068	GUG>UUG	348,268	**AAU>AAU**	429,080
GCG>AUC	787,349	CUU>GCA	308,638	**CAG>CAG**	357,404
GAU>CGC	726,087	CUU>GAA	298,926	**AGC>AGC**	258,564
GCG>AUC	674,901	GAC>GCC	285,894	**CAG>CAG**	225,474
CGA>UCG	635,246	CCU>GAA	283,549	**GAU>GAU**	217,335
GGA>AGC	473,874	CCU>GGG	242,525	CCA>CCG	215,623
GAU>CGC	441,929	UUA>AAA	238,652	**AGC>AGC**	215,121
GCG>CUG	429,895	**GCC>GCC**	238,422	**GGU>GGU**	215,059
AAA>GAG	423,652	GAU>UUG	235,469	**AAG>AAG**	198,659
GCG>AUC	416,940	GCC>GAC	234,454	**GAA>GAA**	198,519

In order to identify the strongest bias in codon-pair contexts they were ranked according to their residual values in Eubacteria, Archaea and Eukaryota. The 10 lowest or highest residuals obtained in each group are shown. Codon-pair contexts that appeared in more than one group are underlined, while codon-pair contexts of identical codons are shown in bold. Eubacteria showed the highest codon-pair biases since the amplitude of the adjusted residuals varied between −309 and 921. Interestingly, 9 out of the 10 highest residuals of eukaryotic ORFeomes corresponded to codon-pair contexts formed by identical codons in both positions (in bold).

**Table 2:** General codon-pair contexts.

Negative codon-pair contexts					
EUBACTERIA		ARCHAEA		EUKARYOTA	
Context	Max.	Context	Max.	Context	Max.
AUG>UAU	−2,443	GCU>AAC	−20,054	ACU>AAG	−36,764
UGG>GCC	0,000	ACU>AAG	−16,118	UCU>AAG	−35,027
AUG>UGA	0,000	ACC>AAA	−13,191	AUU>AAG	−30,44
GUG>UCA	4,769	UGC>GCA	−11,122	AAU>AAG	−27,011
UUC>GCA	8,384	CUC>GAG	−10,371	GCU>AAG	−26,357
GGC>CAA	10,086	ACC>AAG	−9,663	UUU>AAG	−25,784
UUG>UAC	11,826	CAC>AAA	−9,257	CCU>AAG	−25,695
GUU>AGC	20,281	CCC>AAA	−9,050	UGU>AAG	−25,652
GUA>UAC	39,931	UGC>GCU	−6,427	UAU>AAG	−25,582
GCG>UAC	76,191	CCU>AUG	−6,140	AGU>AAG	−25,04
**Positive codon-pair contexts**					
Context	Min.	Context	Min.	Context	Min.
UAC>AAC	−5,974	GAC>UGG	14,515	**AAG>AAG**	67,019
AUG>AGU	−8,173	GGC>UGU	13,185	**GCU>GCU**	51,04
GUU>UCU	−8,426	AAU>CCA	12,265	**GGU>GGU**	34,927
AAA>UAG	−10,902	GGC>UGG	9,454	**AGA>AGA**	29,651
AAU>CCA	−11,288	**UGG>UGG**	8,653	AAG>AAA	28,187
**AGA>AGA**	−0,75	UUC>UGG	8,575	**AAC>AAC**	27,524
AGU>UUU	−5,871	GUA>AAU	7,486	**AGC>AGC**	26,491
AAG>UAA	−6,285	AAC>UGC	6,051	**UCU>UCA**	25,624
GGC>UCU	−18,712	ACA>ACA	5,273	CCU>CCA	23,884
GGG>CAU	−27,619	UGC>CCC	5,243	**CCU>CCU**	23,624

In order to determine whether there are general rules for codon-pair contexts, the contexts that were negative or positive in the highest number of species were identified and sorted by the maximum and minimum residual value found for each context as shown above. As a consequence, contexts that have negative maximum values or positive minimum values have the same sign in all species of each domain (general rules). Codon-pair contexts that appeared more than once are underlined, while codon contexts of identical codons are shown in bold. Major preference for codon repetitions in eukaryotes is clearly visible in the dataset.

### Context preferences exist in coding and non-coding sequences

**Figure 3:**
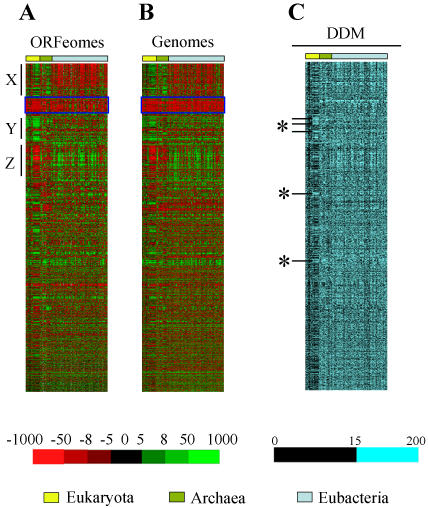
Nucleotide context preferences can be detected in total genome sequences. A large-scale map for codon-pair context was produced using either the ORFeome (panel A) or the total genome (panel B) sequences of 119 species (see Figure 1 and Methods). Such patterns are either universal i.e. present in every species, or visible only in special phylogenetic groups. Surprisingly, most of the ORFeome patterns were also present in total genome sequences, implying that the major forces that drive the evolution of coding sequences are not necessarily connected to mRNA translation. Moreover, when a Differential Map Display (DDM) was built to compare the two former maps (panel C) it became clear that eukaryotes have a more heterogeneous behavior, since they showed greater resemblance between coding and non-coding sequences (darker pattern in the DDM), but they also produced the larger differences found in the DDM (*). These differences correspond either to two-codon context rules imposed by the translational machinery and hence specific of ORFeomes, or to genome biases that are strongly repressed in coding sequences, where they are probably associated to increased decoding error rates. ORFeomes were arranged in the map by domain of life (Eukaryota, Archaea and Eubacteria from left to right) and sorted as shown in Figure S2. Adjusted residuals are colored in the maps so that green cells correspond to preferred and red cells to rejected contexts, while in the DDM major differences (above 15) between residuals of the previous maps are shown in light blue.

A large-scale codon-pair context comparison was carried out to visualize general context patterns, using clustering tools (Figure 3A). Interestingly, a red region, corresponding to negative residual values (rejected context), appeared across the 119 ORFeomes studied (blue box in Figure 3A). These rejected codon-pairs were of the general type NNU_3_-A_1_NN, where N represents any base. Other general patterns in the map represented either preferred codon-pairs in Archaea and Eukarya (Figure 3A; region-Y), rejected codon-pairs in Archaea and Eukarya (Figure 3A; region-Z) or strongly rejected codon-pairs in Eubacteria (Figure 3A; region-X).

In order to evaluate whether those general codon-pair context patterns arose from DNA replication biases, a second large scale comparative map was built using complete genome sequences (coding + non-coding) of the 119 organisms under study. For this, ANACONDA 2.0 scanned full chromosome sequences starting at the first six nucleotides and moved the scanning window three nucleotides at each step. In this way, both coding and non-coding sequences were analyzed and the frequency of all hexanucleotides was computed, without worrying about the DNA strand location or the reading frame of coding sequences, i.e. ORFs were scanned randomly in the frames 0, +1 or +2. This full genome context map (Figure 3B) showed patterns that were also observed in the ORFeome map (Figure 3A), confirming that DNA replication biases strongly influence codon-pair context. Since the difference between full genome (Figure 3B) and ORFeome (Figure 3A) codon context maps could separate global genome biases from translational biases, a DDM was built and the differences between the two were colored using a blue color scale (Figure 3C), as before (Figure 2). The DDM showed significant differences between full genome and ORFeome maps indicating that codon-pair context is also influenced by evolutionary forces that are not related to DNA replication biases. Interestingly, the column corresponding to eukaryotes was generally darker than the rest of the map, meaning that coding and non-coding sequences are similar in eukaryotes (i.e. stronger influence of DNA replication biases). However, the eukaryotic region of the DDM included the highest differences between ORFeomes and genomes (marked with * in Figure 3C), suggesting that the eukaryotic translational machinery also imposes strong selective pressure on specific combinations of codons, resulting in a localized higher divergence between coding and non-coding sequences.

### Codon-pair context is influenced by genome and mRNA translation biases

**Figure 4:**
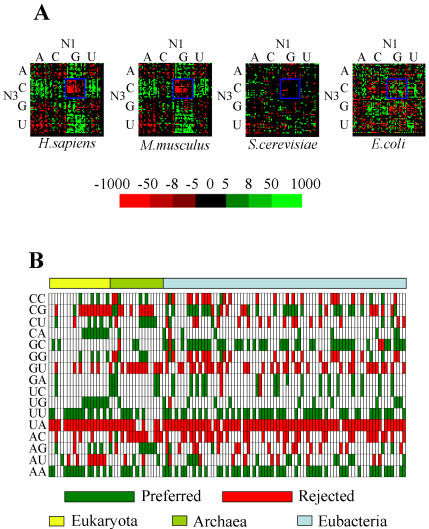
Influence of dinucleotide bias on the codon-pair context preferences. A) In order to highlight the influence of dinucleotide bias on codon-pair contexts, the maps of *H. sapiens*, *M. musculus*, *S. cerevisiae* and *E. coli* were arranged according to their (N_3_-N_1_) context. High degree of context discrimination was achieved by these two positions in higher eukaryotes, especially for the dinucleotide CpG (blue square), however this effect was weak in yeast and *E. coli* showed an opposite preference pattern (green). Adjusted residuals are colored in the maps so that green cells correspond to preferred and red cells to rejected contexts. B) In order to further evaluate the role of the dinucleotide N_3_-N_1_ bias on codon-pair context biases dinucleotide preferences were determined using total genome sequences. The dinucleotide combinations with highest bias were displayed in green (preferred dinucleotides) or red (rejected ones) and correspond to dinucleotides that appear 1% above or bellow the expected level, respectively. The UpA dinucleotide is strongly repressed throughout all domains of life. Other constraints imposed on ORFeomes by genomes biases include the rejection of CpG dinucleotides in higher eukaryotes and the accumulation of CpA and UpG in higher eukaryotes or UpU and ApA in almost all organisms. The last preference is related to high number of tandem repeats of more than 3 consecutive Us or As (Figure S4). ORFeomes were arranged in both maps by domain of life (Eukaryota, Archaea and Eubacteria from left to right) and sorted as shown in Figure S2.

Since DNA replication biases are partly visible at the dinucleotide level [30]–[32], we have constructed individual codon-pair context maps in which rows and columns were sorted to separate P-site codons ending with a particular nucleotide (N3; rows) and A-site codons starting with a particular nucleotide (N1; columns) (Figure 4A). These two consecutive positions of codon-pair context discriminated rather well codon-pair preferences and such discrimination was very strong for high eukaryotes and weak for low eukaryotes and bacteria (Figure 4A). In order to determine whether such dinucleotide bias was linked to translational selection or to overall genome dinucleotide preferences, the dinucleotide bias was determined for the full set of 119 genomes under study (Figure 4B). Overall, rejection of UA dinucleotides in the 3 domains of life was evident; a trend that corresponded to the negative codon-pair context rule (NNU_3_-A_1_NN) described above. The overall dinucleotide biases were also in agreement with the codon-pair context pattern (Figure 4A). For example, the rejection of CpG dinucleotides in higher eukaryotes (with the surprising exception of the honeybee, *Apis mellifera*), was also observed in NNC_3_-G_1_NN codon-pairs (Figure 4A). Other examples were UpG and CpA dinucleotides that were strongly preferred in higher eukaryotes (Figure 4B), a characteristic that was also reflected in codon-pair context maps (Figure 4A). Finally, the dinucleotide biases (Figure 4B) showed overall preference for ApA and UpU dinucleotides. This feature originated from frequent tandem repeats of 3 and more identical bases (Figure S4).

**Figure 5:**
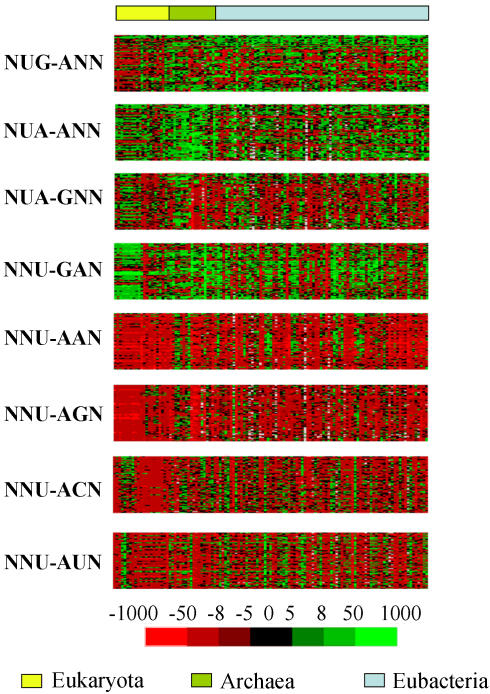
Genome dinucleotide bias has a strong influence on codon-pair context. Since the most generalized negative codon-pair context rule detected corresponds to the general expression NNU_3_-A_1_NN, which includes the out-of-frame translation termination contexts NNU_3_-A_1_A_2_N and NNU_3_-A_1_G_2_N, other out-of-frame context terminators were analyzed separately. For this, the adjusted residuals of such contexts were included in an ORFeome comparison map. It was clear that NNU_3_-A_1_GN and NNU_3_-A_1_AN were indeed the most negative codon-pair contexts bearing out-of-frame stops, followed by NUA_3_-G_1_NN. The other groups of contexts tested did not generate codon-pair context rules, although some of them contained the strongly repressed UpA dinucleotide. The hypothesis that rejection of codon-pair contexts containing out-of-frame stop codons, namely NNU_3_-A_1_A_2_N and NNU_3_-A_1_G_2_N evolved to avoid premature termination was partially contradicted by the existence of similar patterns of NNU_3_-A_1_NN-type contexts that do not include any out-of-frame stops, namely NNU_3_-A_1_C_2_N and NNU_3_-A_1_U_2_N. ORFeomes were arranged in the map by domain of life (Eukaryota, Archaea and Eubacteria from left to right) and sorted as shown in Figure S2. Adjusted residuals are colored in the maps so that green cells correspond to preferred and red cells to rejected contexts.

The only universal rule detected in the large-scale comparison (Figure 3) contained codon pairs of the type NNU_3_-A_1_NN. Since this rule included out-of-frame stop codons, namely UAA or UAG (i.e. NNU_3_-A_1_A_2_N or NNU_3_-A_1_G_2_N), we investigated whether NNU_3_-A_1_NN rejection was related to premature translation termination. For this, we constructed a subset of codon-pair context maps in which the contexts containing out-of-frame stop codons were represented (Figure 5). This approach showed that NNU_3_-A_1_A_2_N and NNU_3_-A_1_G_2_N type contexts were indeed the most negative in almost all ORFeomes. However, NNU_3_-G_1_A_2_N; NU_2_A_3_-A_1_NN and NU_2_G_3_-A_1_NN contexts which also contained out-of-frame stop codons had a majority of positive residual values (green), while NNU_3_-A_1_C_2_N and NNU_3_-A_1_U_2_N contexts that did not contain out-of-frame stop codons had a majority of negative residual values (red). Since some of the positive context rules (Figure 5) included the dinucleotide UpA, which was rejected in the total genomes map (Figure 4), it is likely that dinucleotide bias is not the only cause for the rejection of codon-pair contexts. On the other hand, premature termination was not the only potential problem here, because NNU_3_-A_1_C_2_N and NNU_3_-A_1_U_2_N did not correspond to out-of-frame stop codons and were also strongly rejected in ORFeomes (Figure 5).

### General codon-pair context rules

**Figure 6:**
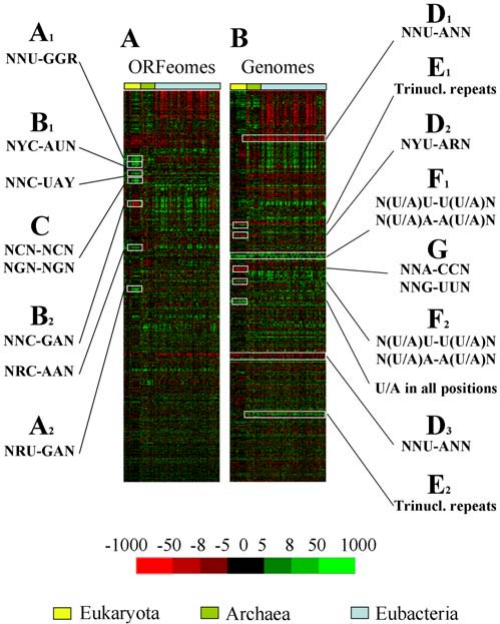
Some codon-pair context patterns are associated to mRNA primary structure biases. A) In order to identify ORFeome specific codon-pair context biases the two large scale context maps were filtered in such a way that only cells that yielded residual differences above 15 between the ORFeomes and total genomes sequences were shown. All other cases were colored in black (see Figure S5 for different display thresholds). Codon-pair context patterns specific of ORFeomes are highlighted on the side of panel A. B) To visualize the patterns that appear in genomes and are absent in ORfeomes, large-scale comparative maps obtained with total genomes and ORFeomes were subtracted and only the cells that yielded differences above 15 were displayed. This highlighted patterns that are strongly preferred or repressed in coding sequences and may correspond to mistranslation hot spots.

In order to highlight the codon-pair context preferences that were exclusive of coding sequences, the original map of ORFeomes (Figure 3A) was rebuilt to show in black cells whose residuals values were similar to those of identical contexts in the complete genomes map (Figure 3B). In this filtered map (Figure 6A) green and red colored cells corresponded to those context residual values calculated for ORFeomes that were significantly different from those calculated for complete genomes, i.e. cells that were colored in blue in Figure 3C. This large-scale comparative map allowed extraction of ORFeome specific codon-context patterns, while the converse filtering originated a complete genomes map that permitted extraction of genome specific patterns (Figure 6B). This approach highlighted clear codon-pair context differences between ORFeome and complete genome maps (Figure 6A,B). Interestingly, these patterns corresponded to different sets of codon-pair contexts that could be easily described by the expressions annotated on the side of each map (Figure 6A,B see also Figure S5A,B for different thresholds of visualization). In almost all cases, these codon-pair context rules fixed the last nucleotide of the first codon and the first nucleotide of the second codon, confirming that (N_3_-N_1_) positions shape codon context. Remarkably, the major patterns that appeared in the filtered map for complete genomes (Figure 6B) were related to UpA-rich hexanucleotides that produce weak codon-anticodon interactions in coding regions and should hence be under differential selective pressure in both types of sequences.

## Discussion

Mistranslation is a poorly understood biological phenomenon which is influenced by various protein synthesis factors and mRNA primary structure features [13], [33], [34]. In order to shed new light on how the later influences decoding error and extend previous studies carried out mainly on the effect of codon usage on mistranslation [25], [35], we are investigating the effect of codon-pair context on decoding fidelity. Our comparative genomics approaches unveiled the effect of both genome replication and translation specific biases on codon-pair context. The few studies carried out to date on codon-pair context were unable to distinguish those two types of biases [12], [36]–[38]. Our large scale approach confirmed the importance of genomic biases but also unveiled important translational biases that shape codon-pair context and should be primary targets for mistranslation hot spots.

Large-scale genomic analysis, such as the one that we have performed, allows for obtaining a global view of mistranslation in a way that is totally out of reach from analysis of single ORFeomes. Indeed, comparison of large sets of codon-pair context data unveiled the main codon-pair context patterns that exist in the 3 domains of life. Interestingly, when the most preferred or repressed codon-pair contexts of all organisms were considered (Table 1), but also when common rules were selected (Table 2), there was little or no overlapping between the context patterns of the 3 domains of life. This suggests that genome replication and/or mRNA translation in each domain imposes specific constraints to decoding sequences which produce different codon-pair context outcomes. Also, the phylogeny of individual species appeared as an important determinant of its codon-pair context behavior (Figure 2), in a similar manner to that described for codon usage bias [39] or dinucleotide genome signatures [31], [32].

### Influence of genome wide biases on codon-pair context

Our observation that ORFeomes and total genomes produce similar patterns of codon-pair context (Figure 3) confirmed previous studies [4], [40], [41]. This implies that most sequence constraints that can be detected in coding sequences are not imposed by the translational machinery, but arise from selective pressure imposed by DNA replication and related biases. That codon usage biases were mainly due to mutational pressure and only secondarily to translational selection further confirmed the relevance of DNA replication biases on codon-pair context [7]. In this scenario, one is prompted to hypothesize that the translational process may work with sub-optimized mRNA sequences since codon-context fine tunes decoding fidelity [15], [22], [23].

Genomes are known to have biased dinucleotide frequencies [31], a feature that has frequently been used to produce genomic signatures of phylogenetical and taxonomical relevance [31], [32]. At the ORFeome level this bias influences codon usage [32] but may also interfere with codon-context, whenever the last nucleotide of one codon is associated with the first nucleotide of the second codon of the pair. Indeed, (N_3_–N_1_) contexts explained part of our results (Figure 6) and confirmed the good discrimination obtained when one ORFeome map for codon context was arranged according to the last position of the first codon and the first position of the next codon (Figure 4A).

The association bias of two consecutive nucleotides is a characteristic of genomes which results from global selective pressures acting upon DNA at the level of repair and replication mechanisms [32] or ecological constraints that may influence, for instance, the overall G+C content of the genome [42]–[44]. Regulatory activity acting upon the entire genome is another cause of dinucleotide bias. CpG dinucleotides, for example, are signals for DNA methylation, a mechanism commonly used by higher organisms to protect their genome from selfish DNA elements and to regulate gene expression [5], [6]. Our dinucleotide bias analysis for the 119 organisms confirmed a clear rejection of CpG methylation in coding sequences of high eukaryotes, as would be expected, since methylated DNA becomes unavailable for transcription and hence translation [5]. On the other hand, UpA dinucleotides are highly repressed in DNA sequences of most organisms [7], [31], [45], [46]. Interestingly, UpA dinucleotides are sites for preferential hydrolysis of RNA by macrophage ribonucleases [45] destabilizing RNA molecules [7] and should hence be avoided [45]. Furthermore, Duan and colleagues [7] proposed that mRNA stability imposes strong selective pressure on synonymous codon usage and it is likely that this is also true for codon-pair context. Our data confirmed that hypothesis since NNU_3_-A_1_NN contexts were highly repressed in the 119 different genomes analyzed.

### Influence of translational biases on codon-pair context

As already mentioned, the unique universal rule that could be detected in the 119 genomes analyzed was rejection of most codon-pair contexts of the type NNU_3_-A_1_NN (Figure 3). Clearly, this trend is a direct result of repression of the TpA dinucleotide in total DNA sequences (Figure 3). However, it was surprising that other UpA bearing contexts did not show strong rejection. For example, NU_2_A_3_-A_1_NN contexts are mainly preferred in coding sequences (Figure 5) indicating strong differences between codon-pairs containing UpA dinucleotides and suggesting that translation does influence codon-pair choice.

When a large-scale comparison of codon-pair context excluded global genome biases (Figure 6A) it became evident that contexts that were truly produced by translation-driven selection were grouped in negative or positive rules depending on the phylogeny of the organisms. This was in agreement with the previous observation that strongly biased codon-pair contexts were different between the 3 domains of life (Tables 1, 2), and supported the hypothesis that differences in the translational machineries of different organisms reshape mRNA primary structure in different ways. For example, the NNC_3_-N_4_NN contexts pattern of higher eukaryotes (B1 and B2 in Figure 6A) could be explained by specific decoding rules of C-ending codons in Eukarya. Indeed, eukaryotic species translate several C-ending codons by wobble pairing rules using inosine [47], which recognizes A, C, or U at the wobbling position [48] while bacterial species decode most C-ending codons with Watson-Crick C-G base pairing between codon and anticodon [47].

As to the other minor rules highlighted on the left side of Figure 6, namely NNU_3_-G_1_G_2_R, NRU_3_-G_1_A_2_N and NG_2_N-NG_2_N or NC_2_N-NC_2_N, they may be related to both canonical decoding of U-ending codons in eukaryotes (A1 and A2 in Figure 6A) and to the existence of runs of special sets of amino acids, namely serine/proline/threonine/alanine and arginine/glycine (C in Figure 6A). That contexts of repeated codons are preferred in eukaryotic genes (Tables 1,2) and that proline, alanine and glycine are frequently found in amino acid runs of human genes [49] corroborates the above hypothesis.

On the other hand, most of the major genomic constraints that were not present in coding sequences, namely NNU_3_-A_1_NN, NYU_3_-A_1_RN and N(U/A)_2_U_3_-U_1_(U/A)_2_N or N(U/A)_2_A_3_-A_1_(U/A)_2_N rules (Figure 6B) were associated to weak decoding interactions involving A-U codon-anticodon pairing. Moreover, these rules are produced by either strong genomic dinucleotide bias against UpA (Figure 6B, rule D) or by rejection of error prone UA-rich codon-pair contexts in coding sequences (Figure 6B, rule F), in a clear confirmation of the additive effect of translational and non-translational selective pressures. Finally, we could also see a preference for trinucleotide repeats in non-coding sequences that was not detectable in coding regions, at least in eukaryotes (Figure 6B, rule E). This has already been described in primates and is related to strong mRNA primary structure constraints associated to high mRNA decoding efficiency [50].

### Conclusions

Codon-pair contexts are biased in ORFeomes and such bias is the result of both translation and non-translation driven processes. Indeed, translational and DNA replication/repair and cis regulatory elements act synergistically on codon-pair context. This myriad of selective pressures creates significant difficulties to the identification of codon-context biases associated to mRNA translation only. Our large scale comparative genome approach indicated that: i) there is a strong influence of non-translational selective pressures upon coding sequences, especially in eukaryotic organisms since these have a higher degree of resemblance between ORFeome and total genome biases; ii) the strongest non-translational selective pressures that could be identified were dinucleotide biases, mainly imposed by regulatory cis-elements linked to DNA methylation or mRNA stability [5], [45], and preference for trinucleotide repeats, usually associated with DNA polymerase slippage during replication [51]; iii) apart from this non-translational noise, DNA coding sequences showed specific features that could be related to mRNA translation, namely repression of usage of premature termination or error-prone contexts associated to weak codon-anticodon interactions. It will now be most interesting to validate these *in silico* data *in vivo*, and identify experimentally the codon-pair contexts that are strongly selected for high mRNA decoding fidelity.

## Methods

### Primary data sources

Nucleotide sequences, of complete genomes and assembled ORFeomes, were downloaded from GenBank or Ensembl Web sites (Genbank: ftp://ftp.ncbi.nih.gov/genomes/; Ensembl: ftp://ftp.ensembl.org/pub/) between December 2005 and January 2006. These included the DNA sequences of 81 eubacterial, 18 archaeal and 20 eukaryotic species. Plasmid sequences were not included in the analysis and all chromosomal sequences from one genome were analyzed together by ANACONDA 2.0. The total set of files downloaded and used in this study is documented as supplementary data (Figure S2).

### Statistical analyses

Two-codon context bias was studied in complete ORFeome sequences using the residual analysis tools available in the software package ANACONDA 1.0 (a detailed explanation of this software can be found in [20], [29]. ANACONDA is publicly available at http://bioinformatics.ua.pt/submited-papers).

Briefly, this methodology counts all consecutive pairs of codons and uses statistical analysis for contingency tables where a multinomial distribution is assumed (Figure 1B). The final result of such statistical approach is the calculation of adjusted residuals for each codon pair present in any ORFeome. The adjusted residuals give direct information about preference or rejection of these codon pairs in relation to what would be expected assuming independence of the distribution (Figure 1B).

Since, under independence between two consecutive codons, the adjusted residuals *d_ij_* have a standardized normal probability distribution [52], we have concluded that: 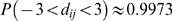, as the total number of observations is very high. This means that, for a 99,73% confidence level, an adjusted residue was statistically significant if its absolute value was greater than 3 [20]. However, this approach was based on a local analysis for each residual value. Herein, we considered a global analysis for each species and have thus constructed a simultaneous confidence region for all residual values. Since there are K = 61×64 different intervals we have introduced the Bonferroni correction to ensure an overall level of significance of α (usually α = 0.05, 0.01, 0.001). The Bonferroni correction is used for correction where each interval is constructed at a 100×(1–α/K) level (see, for example, [53]). Therefore, a–*a* to *a* interval at a confidence level of 100×(1−α/(61×64)) was constructed for each adjusted residual value *d_ij_*. Considering again the asymptotic normal distribution of *d_ij_* [52] we had *a*≈4,70341 when 1–α = 0,99, *a*≈5,15350 when 1–α = 0,999, *a*≈8,16204 when 1–α = 0.01×10^−10^. Thus, we assumed that the codon-pair adjusted residuals that fall within the interval −5 to 5 were not statistically significant, for a global confidence level of 99% (colored in black in all maps shown herein).

The final output of residual analysis performed by ANACONDA is a codon-pair context map for each ORFeome being studied (Figure 1C). These maps show one colored square for each codon-pair, the first codon corresponding to rows and the second corresponding to columns in the map. The color scale chosen for this layout determines that preferred contexts are shown in green while repressed ones appear in red (Figure 1C).

Taking advantage of the automated statistical analysis performed by ANACONDA, individual maps for all 119 ORFeomes were built (see Figure S3). In order to facilitate large-scale comparison of maps these were converted into single lines and clustered together (Figures 1D,E and 3). The patterns that appear in the resulting comparative map were then characterized by the codon-pair contexts that were present in each pattern. Also, the values of the adjusted residuals calculated for each species were corrected for ORFeome size to allow direct comparisons among ORFeomes.

The above approach was also used to study total genome sequences of the same 119 species in order to differentiate between the effect of translational selection acting upon coding sequences alone and genome mutational biases. With the same purpose, the bias for dinucleotides was studied in total genome sequences, and shown as observed frequencies, colored in green or red whenever the result was 1% above or below the expected value, respectively (Figure 4B).

## Supporting Information

Figure S1 Data normalization. In order to correct the size differences of ORFeomes, particularly between eukaryotes and non-eukaryotes, the adjusted residuals were normalized for 21 million codons which correspond approximately to the larger ORFeome analyzed (X. tropicalis). Normalization of codon-pair data for human chromosomes 1, 2, 3, 22 and ORFeome are displayed. The normalization effect is shown by the brightness of the maps, which is variable in non-normalized maps (above) and constant in normalized ones (below). After data normalization the differences between maps could be compared as shown in the DDM (right end of the Figure).
(4.32 MB TIF)

Figure S2A List of species used. All species used in the study are listed according to the download order. The database of origin and respective accession numbers are indicated. A - Eukaryotes; B - Archaea and Eubacteria; C - Eubacteria (cont.).
(0.54 MB TIF)

Figure S2B (0.54 MB TIF)

Figure S2C (0.54 MB TIF)

Figure S3A Individual codon-pair context maps of the 119 species. The codon-pair context maps built with ANACONDA software for individual ORFeomes are shown as ordered in Suppl. Figure S2.
(4.32 MB TIF)

Figure S3B (4.32 MB TIF)

Figure S3C (4.32 MB TIF)

Figure S3D (4.32 MB TIF)

Figure S3E (4.32 MB TIF)

Figure S3F (4.32 MB TIF)

Figure S3G (2.16 MB TIF)

Figure S3H (2.16 MB TIF)

Figure S3I (2.16 MB TIF)

Figure S3J (2.16 MB TIF)

Figure S4 A and U bases are preferentially arranged in polynucleotide strings. In order to check if the preference detected for AA and UU dinucleotides in total genomes (Figure 4B) was due to a tendency for these bases to appear as polynucleotide strings we counted the number of times each individual base appeared isolated or in strings of two, three or more equal bases. The result of this approach points to a clear positive bias towards the accumulation of 3 or more consecutive A or U bases in total genomes.
(0.10 MB TIF)

Figure S5A Codon-pair context patterns that are exclusive of ORFeomes or genomes. The filtering technique that was used to determine the biases of codon-pair contexts in coding and total sequences (Figure 6) was further explored in here to evaluate the strength of those biases. This was done by gradually increasing the threshold of the residuals (D) that are significantly different in both maps, i.e. those that were allowed to appear in their original colors in the filtered map. When D was increased, only major differences between ORFeomes and genomes were visible, corresponding to differences between the residuals of ORFeomes and genomes maps that stay above 15, 20, 30 or 50. The strongest rules detected by this approach correspond to those identified as B1 and B2 in the filtered map for ORFeomes (map A in Figure 6), and F1, F2 and E2 in the filtered map for genomes (map B in Figure 6), because they are still visible when D = 50.
(0.55 MB TIF)

Figure S5B (0.53 MB TIF)

Table S1 Codon-pair distribution similarities between the 3 domains of life. In order to compare the overall distribution of codon-pair contexts among the 119 organisms we have calculated the Spearman's correlation coefficients between all pairs of ORFeomes, producing a triangular colored map. The 119 species were organized by domain of life and sorted alphabetically in each domain. Pairs of species that were not statistically correlated (for a level of significance of 5%) are colored in grey, while green colored cells indicate pairs of species that were highly correlated (correlation coefficient above 0,80), and blue colored cells correspond to the major values fount inside each domain.
(0.32 MB XLS)
